# High-energy, high-resolution, fly-scan X-ray phase tomography

**DOI:** 10.1038/s41598-019-45561-w

**Published:** 2019-06-20

**Authors:** Hongchang Wang, Robert C. Atwood, Matthew James Pankhurst, Yogesh Kashyap, Biao Cai, Tunhe Zhou, Peter David Lee, Michael Drakopoulos, Kawal Sawhney

**Affiliations:** 1 Diamond Light Source Ltd, Harwell Science and Innovation Campus, Didcot, OX11 0DE UK; 2 Research Complex at Harwell, Rutherford Appleton Laboratory, Harwell, Oxfordshire OX11 0FA UK; 3 School of Earth and Environment, University of Leeds, Leeds, LS29 9ET UK; 4 Instituto Tecnológico y de Energías Renovables (ITER), 38900 Granadilla de Abona, Tenerife, Canary Islands Spain; 5 Instituto Volcanológico de Canariaes (INVOLCAN), INtech, La Laguna, Calle Rectora María Tejedor Salguero, 35, 38320 San Cristóbal de La Laguna, Tenerife, Canary Islands Spain; 6 Technical Physics Division, Bhabha Atomic Research Centre, Mumbai, 400085 India; 7 School of Metallurgy and Materials, University of Birmingham, Birmingham, B15 2TT UK; 8 Department of Mechanical Engineering, University College London, Torrington Place, London, WC1E 7JE UK

**Keywords:** Imaging techniques, Geology

## Abstract

High energy X-ray phase contrast tomography is tremendously beneficial to the study of thick and dense materials with poor attenuation contrast. Recently, the X-ray speckle-based imaging technique has attracted widespread interest because multimodal contrast images can now be retrieved simultaneously using an inexpensive wavefront modulator and a less stringent experimental setup. However, it is time-consuming to perform high resolution phase tomography with the conventional step-scan mode because the accumulated time overhead severely limits the speed of data acquisition for each projection. Although phase information can be extracted from a single speckle image, the spatial resolution is deteriorated due to the use of a large correlation window to track the speckle displacement. Here we report a fast data acquisition strategy utilising a fly-scan mode for near field X-ray speckle-based phase tomography. Compared to the existing step-scan scheme, the data acquisition time can be significantly reduced by more than one order of magnitude without compromising spatial resolution. Furthermore, we have extended the proposed speckle-based fly-scan phase tomography into the previously challenging high X-ray energy region (120 keV). This development opens up opportunities for a wide range of applications where exposure time and radiation dose are critical.

## Introduction

X-ray computed tomography (CT) allows for visualizing interior features of a sample in a non-destructive way. Distinctive features can be identified if material composition and density vary significantly. According to the Beer-Lambert law, X-ray penetration is an exponentially decreasing function of sample thickness. It follows that the reduced photon flux will inevitably lead to poorer absorption contrast for thicker samples. One fundamental X-ray interaction with matter is the photoelectric effect, which relates to X-ray beam absorption. Another is Compton (incoherent) scattering, and a third is coherent scattering, which is the primary process responsible for diffraction^1^. The probability of photoelectric absorption is approximately proportional to (Z/E)^2^, where Z is the material atomic number and E is the photon energy. As the photon energy E increases, the likelihood of interaction between X-ray and matter through the photoelectric absorption process drops rapidly, while Compton scattering dominates at higher energies. It follows that higher energy (>50 keV) X-rays can penetrate deeply into materials and result in lower absorbed radiation dose. With increasing X-ray energies, however, the reduced absorption cross section for materials leads to lower contrast in absorption images.

An important property of X-rays and their interaction with matter is that the real part (phase) of the refractive indices of many materials in the X-ray range is significantly higher than the imaginary part (absorption). This means that X-ray phase-contrast CT has the potential to provide enhanced image contrast compared to absorption CT. Over the last few decades, diverse X-ray phase imaging techniques have been developed and several of them have been demonstrated to be compatible with the use of high energy X-rays. For example, analyzer-based phase contrast imaging has been applied with X-ray energies up to 60 keV^3^, however, this technique requires a highly parallel X-ray beam with high temporal coherence. Although both the edge illumination technique and grating interferometry have been demonstrated to achieve phase signals and quantify X-ray refractive indices above 85 keV^2,4^, the fabrication of precision gratings or masks with the large aspect ratios that are required for high energy X-ray beams imposes a major technical challenge. Although no optics are required for in-line X-ray phase contrast imaging^5^, the stringent requirement for a highly spatially coherent source and *prior knowledge* about the sample limits its widespread application.

Recently, the speckle-based imaging technique has been developed to overcome these challenges^6–10^. It uses inexpensive materials (such as sandpaper or steel wool) as wavefront modulators and does not require complex experimental setup. Moreover, it provides quantitative and multimodal images (absorption, phase and dark-field) and permits either high speed by using a single-shot tracking mode or high spatial resolution with multi-frame scanning mode. In the single-shot tracking mode, the phase information can be extracted from a single speckle image. However, the spatial resolution is deteriorated due to the use of a large correlation window to track the speckle displacement. In scanning mode, the modulator steps across the beam. One frame of an image is captured at each position. In this mode, the correlation is performed between the data obtained at each step, rather than over a large correlation window. This means the spatial resolution is limited by the speckle size and detector pixel size, instead of the correlation window size. Several new scan schemes have been developed recently which choose a compromise between scan steps and angular sensitivity^11,12^. Regardless of how this compromise is reached and for what purpose, all of these schemes still require multi-frame scanning routines. The multi-frame scan is typically performed in a step-scan mode, which requires the detector to wait until the stages have moved, and settled, at the target position before data acquisition can re-commence.

The time overhead of the step-scan accumulates over a large number of scan positions. This accumulation severely limits the speed of data acquisition for scans such as tomography, which require hundreds or even thousands of projections. Prolonged acquisition time also increases risk associated with the stability of experimental instruments and radiation damage to the samples. Therefore, it is desirable to decrease acquisition time for X-ray phase tomography to speed up the experiments and open up new avenues of research.

In this work we report a fast phase contrast CT method based on the speckle scanning technique, operating at high X-ray energies up to 120 keV. The technique employs a fly-scanning scheme resulting in more than an order of magnitude reduction in the acquisition time and the delivered dose, compared to the existing step-scan methods. Furthermore, these gains are achieved without compromising spatial resolution and the quality of the phase tomography. This development represents an important milestone in quantitative phase contrast CT, especially at high X-ray energies.

**Figure 1:**
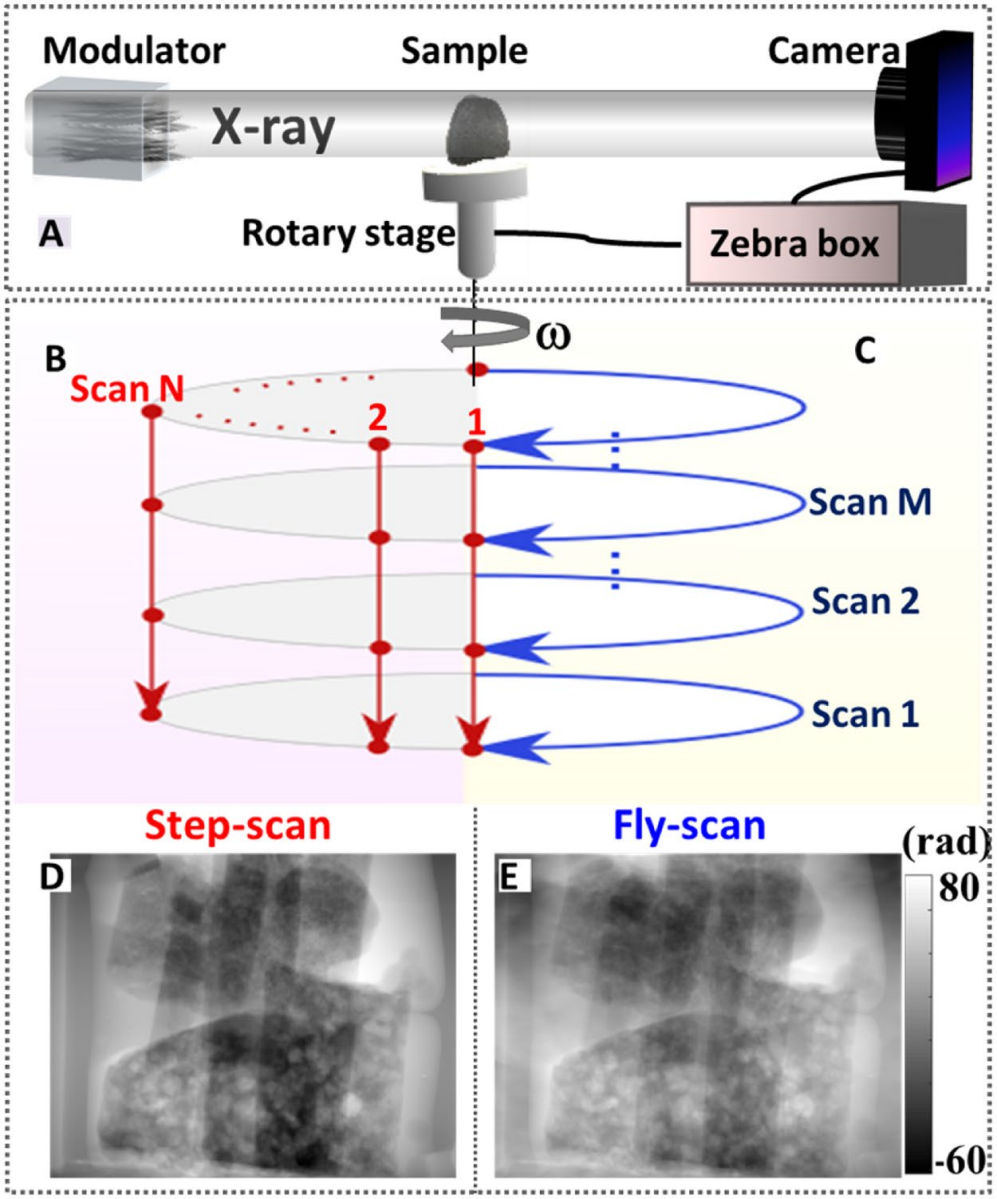
(**A**) Schematic illustration of the experiment setup at I12 beamline. The fly-scan scheme is implemented with a Zebra box control system by synchronizing the rotary stage position and camera triggering signal. (**B**) For speckle-based phase tomography with the standard step-scan scheme, the sample is held stationary while the modulator is stepped through M positions across the beam, and the modulator will be repeatedly scanned when the sample is rotated at all the projections N. (**C**) For the proposed fly-scan scheme, the tomography rotation is performed continuously with projection N at different modulator position M. (**D**) The reconstructed phase images from one projection by using the step-scan scheme. (**E**) The reconstructed phase images taken by using the fly-scan scheme with the same parameters.

The technique has been demonstrated at the I12 beamline of the Diamond Light Source (DLS), UK^13^. Figure 1(A) shows the experimental setup with steel wool as the wavefront modulator, a sample, and an X-ray camera. As shown in Fig. 1(B), for the standard step-scan method^14^, the modulator will be scanned over M = 100 steps with a step size of ν = 5 µm. In this standard scheme, the sample is held stationary while the modulator is stepped through M positions. Then the sample is rotated one increment (of 0.15°), and the mask is again stepped through its M positions. In this way the sample is in precisely the same position during the stepping. For N projections, the time overhead of starting and stopping the mask is repeated M*N times, and many precise motors take hundreds of milliseconds for each motion. Although the exposure time for each step was only t = 5 ms, the total acquisition time for those 100 scan steps was 76 s due to the time overhead. The total time required for a tomography scan with N = 1200 projections with orientation angles ranging from 0° to 180° would hence normally be more than one day (T = 25 hours).

The fly-scan mode^15^ (Fig. 1C) achieves a shorter time overhead than the step-scan mode by using a ZEBRA system, which simplifies the connection and generation of trigger and gate signals in tomography experiments, and also directly reads the rotation stage position encoders. This allows capture of the stage position at the time the camera is triggered for each exposure. It also allows trigger signals to be sent to the camera when the sample reaches specified angles. The sample rotation is performed in between modulator steps, saving all the time overhead of moving and stopping the rotation stage. Therefore, the time overhead for the rotation scan has been significantly reduced. The total acquisition time of the fly-scan scheme was less than 2 hours, while the step-scan scheme performed under the same conditions took over 25 hours.

Figure 1 (D,E) are the retrieved phase images of a single projection from the conventional scheme and the proposed fly-scan scheme performed with the same number (M = 100) of modulator positions. Both methods provide similar information as expected. The standard deviation calculated from the empty space in the phase image from the fly-scan scheme is 2.6 times higher than that of the step-scan scheme. The higher noise experienced using fly-scan scheme is mostly due to slow changes of the speckle pattern during the entire phase tomography scan (2 hours), while such changes are negligible for single step-scan in short period (76 s).

**Figure 2:**
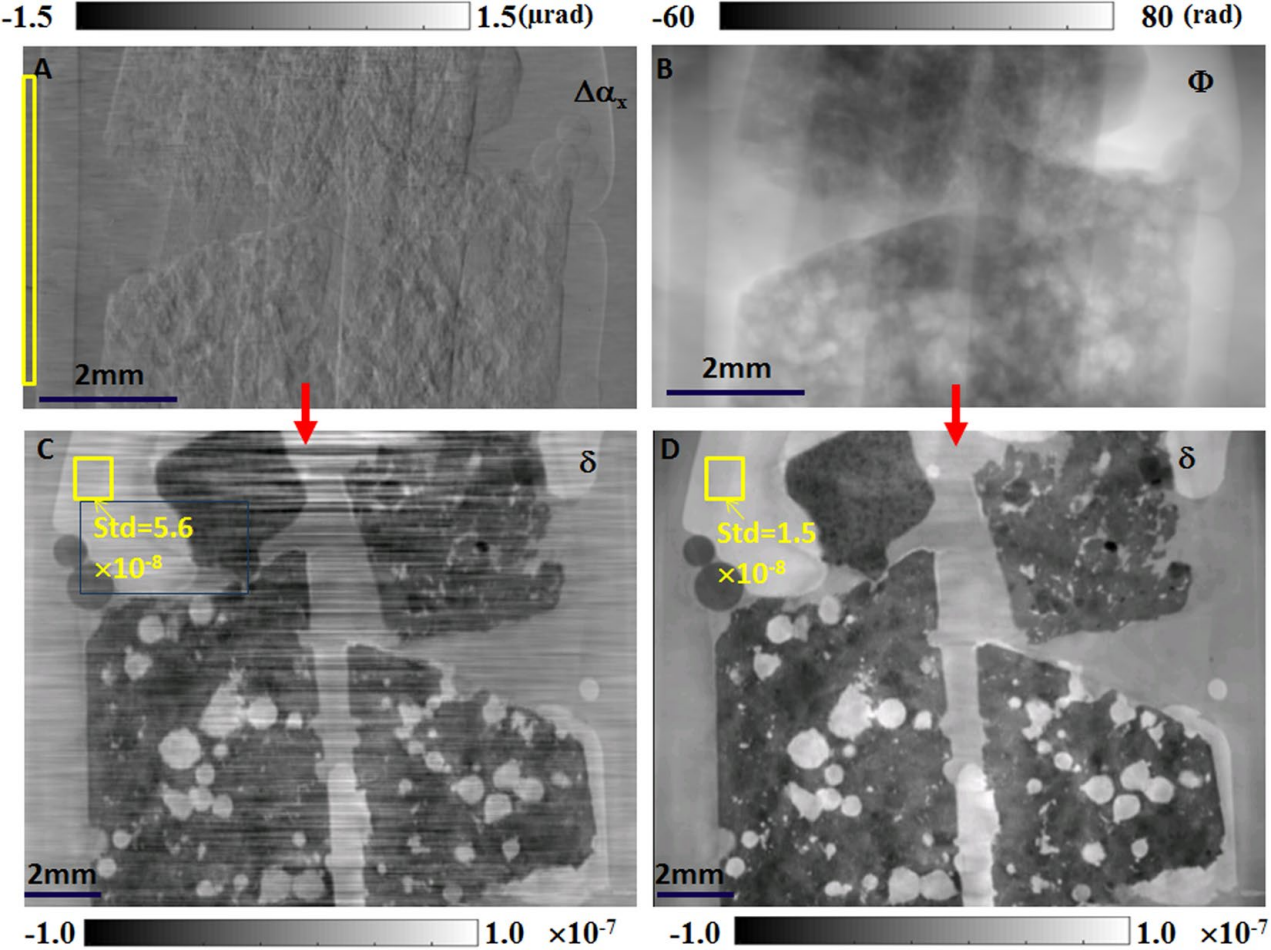
Retrieved results for a rock sample using fly-scan phase tomography. (**A**) Horizontal phase gradient and (**B**) reconstructed phase map of one projection. The standard deviation within the empty space in the yellow rectangular box is used to quantify the angular sensitivity of the wavefront gradient. (**C**,**D**) Sagittal slices of the reconstructed phase tomography using (**A**) the phase gradient and (**B**) the phase shift, respectively. The standard deviation in the paraffin wax inside the yellow square box is much higher in (**C**) (5.6 × 10^−8^) than in (**D**) (1.5 × 10^−8^) due to the stripe artefacts. The scale bar at the bottom left is 2 mm.

To explore the potential applications of the proposed fly-scan phase tomography to geological science, a few different types of volcanic rocks were measured at 53 keV in the first case study. Volcanic rocks are multiphase materials that exhibit highly inhomogeneous texture, chemical composition, grain sizes and phases. Here picrite, andesite, rhyolite and obsidian are selected because they span a wide range of properties. Horizontal phase gradient and reconstructed phase map of one projection is shown in Fig. 2(A,B), respectively. As shown in Fig. 2(C), the phase image reconstructed from the phase gradient contains stripe artefacts, which decrease the quality of obtained datasets and often impede the subsequent analysis. Using only the horizontal phase gradient will inevitably lead to severe stripe artefacts in the image along the horizontal direction of integration. The stripe artefact issue is well-known in the grating interferometer technique^16^, which only provides the phase gradient perpendicular to the grating’s lines. Although some advanced algorithms have been developed to suppress or reduce them, the full removal of such stripe artefacts still remains challenging^17^. From this perspective, the speckle-based technique bears a unique advantage over the 1D grating-based technique, since the two-dimensional phase gradient images can be simultaneously retrieved from a scan along a single direction. As described in Eq. (2), phase projection images were integrated from the horizontal and vertical phase gradients. The direct integration from single gradient images is avoided, as illustrated by the lack of stripe artefacts in Fig. 2(D). It should be noted that the reconstructed phase projection images sometimes contain low frequency noise, which should be removed before the back projection is used. In addition, the standard deviation of the selected region of the phase-reconstructed slice in Fig. 2(D) is Δδ = 1.5 × 10^−8^, which is more than three times better than in the reconstruction from the horizontal phase gradient, Fig. 2(C) (Δδ = 5.6 × 10^−8^). This underscores the advantage of using phase projection images to reconstruct the refractive index decrement. Key information can then be gained from imaging the large numbers of crystal boundaries that normally occur in materials of moderately high density^18^.

The single-shot speckle tracking scheme was first proposed when X-ray near-field speckle imaging was invented^6,7^. A subset of image data (25 × 25 pixels) surrounding each pixel from the sample speckle image is chosen, and a digital image correlation (DIC) is employed to track the speckle displacement between the sample speckle image and reference speckle image. Since only a single speckle image for each projection is required, fast and low-dose phase contrast CT has been implemented with the speckle tracking scheme^19,20^. To demonstrate the improved spatial resolution inherent in the multi-frame speckle scanning technique, the fly-scan tomographic images at the first modulator position were used and processed using the speckle tracking approach.

**Figure 3:**
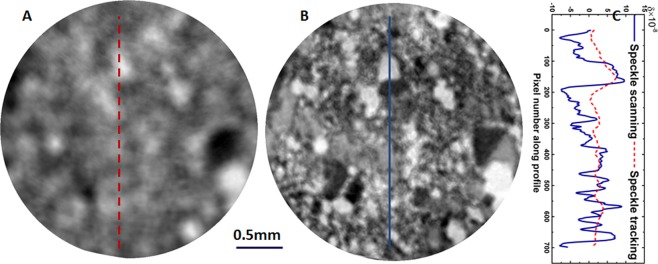
Reconstructed phase contrast axial slices from tomographic reconstruction of volcanic rock sample using (**A**) single-shot speckle tracking and (**B**) multi-frame speckle scanning technique. (**C**) Line profile of the retrieved refractive index decrement. The scale bar at the bottom is 0.5 mm long.

Figure 3 shows one phase slice of a volcanic rock after reconstruction using both of the above techniques. For the single-shot speckle tracking scheme, the spatial resolution is inherently limited by the correlation window size, hence the small crystals of the volcanic rocks cannot be resolved in Fig. 3(A). The spatial resolution for the single-shot speckle tracking scheme is about 60 µm with the effective pixel size of P = 3.5 µm. By comparison and as illustrated in Fig. 3(B), all these features can be clearly resolved in the reconstructed image using the multi-frame speckle scanning approach. The spatial resolution for the speckle scanning approach is only about 7 µm since only a few pixels were selected for pixel-wise data processing. The corresponding line profiles between the two schemes are marked with the dashed red line (A) and blue solid line (B) and shown in Fig. 2(C) which demonstrates that details are visible in the line profiles using the speckle scanning approach, while they are convolved by the correlation window used by the speckle tracking scheme.

For the conventional speckle step-scan technique, the same number of sample speckle images and reference speckle images were recorded with and without the sample in the X-ray path^21^. The DIC is then performed between the retrieved sample speckle signal and reference speckle signal for each pixel. In practice, the window size of the sample speckle signal is smaller than the one of the reference speckle signal^22^. For M = 100 reference speckle images, the offset O = 10 images for both start and stop positions have been removed for the sample speckle signal. Thus it only requires to collect M-2 × O = 80 sample speckle images for data analysis for each projection. As a result, the acquisition time can be reduced by 20% for the present case.

**Figure 4:**
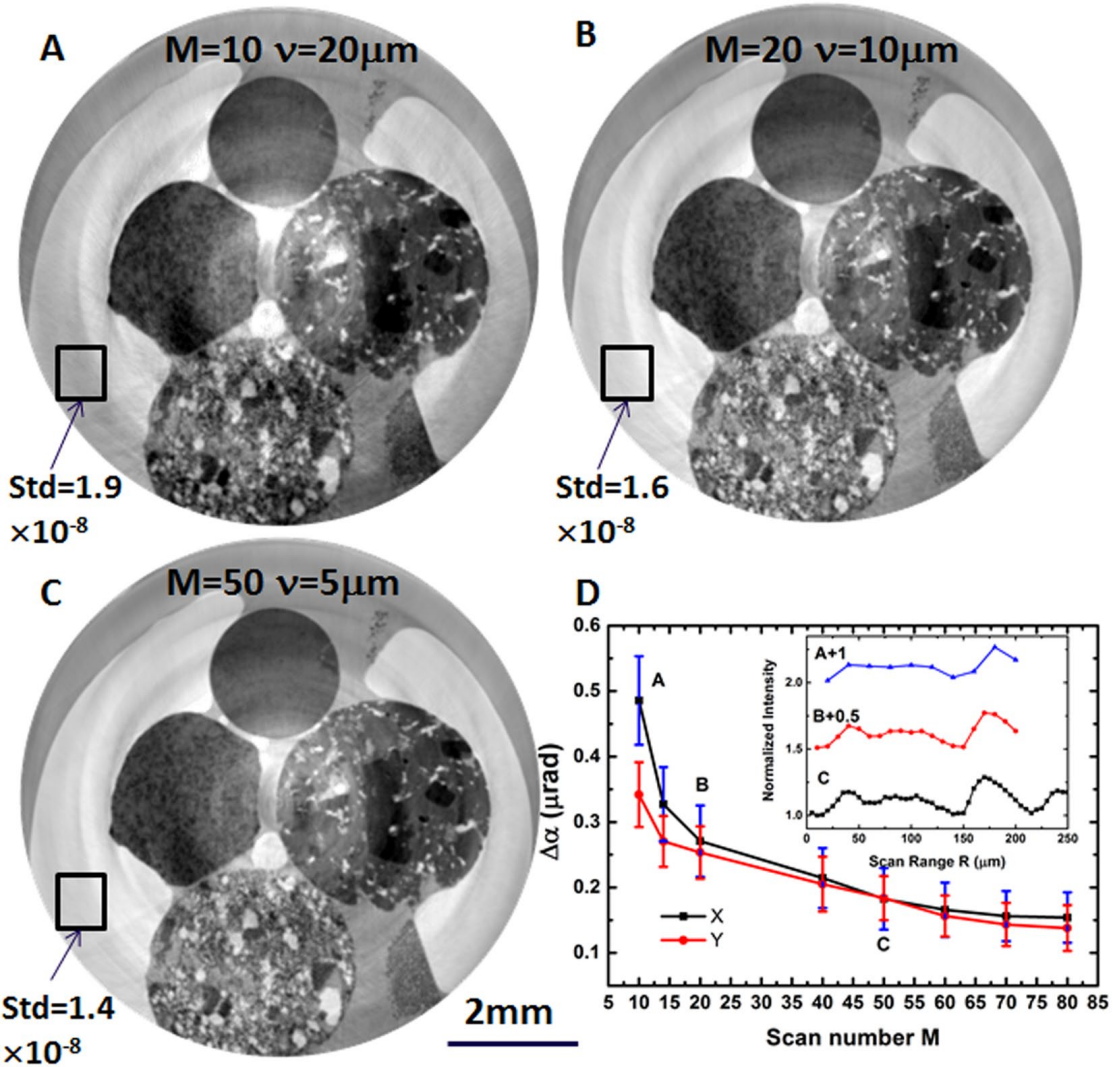
Comparison of reconstructed phase slice with different numbers of modulator scans (**A**) M = 10, (**B**) M = 20 and (**C**) M = 50 using the proposed fly-scan tomography. (**D**) The angular sensitivity as function of modulator scan number M. Inset of (**D**) is the speckle normalized intensity as function of M, the number of scans. The scale bar corresponds to 2 mm. Top sample is fine-grained rhyolite, left is coarser rhyolite, right is picrite, bottom is andesite.

The high spatial resolution is achieved by the speckle scanning approach at the cost of a large number of scanned speckle images for each projection. For the proposed fly-scan tomography, the sample is continuously rotated at each wavefront modulator position. Hence, the total acquisition time is linearly proportional to the number M of modulator positions. For the speckle scanning technique, the step size ν is usually chosen to be smaller than the averaged speckle size ς, while the total scanning range R = M × ν should be some ~2–4 times the maximum speckle size for the best compromise between sensitivity and acquisition time. The number M of modulator positions should be chosen as small as possible in order to further reduce the data acquisition time for the phase CT. For the present case, the diameter of steel wool is about 25 µm, and the averaged speckle size generated by the steel wool is about ς ≅ 80 µm. To further reduce the speckle size and the scanning range, the steel wool can be potentially replaced with porous engineering materials^23^ tailored to produce suitable speckle. One speckle intensity curve as a function of R is shown in the inset of Fig. 4(D), in which the scan number and step size are (A) M = 10, ν = 20 µm (B) M = 20, ν = 10 µm and (C) M = 50, ν = 5 µm. The angular sensitivity of the speckle scanning technique is mainly dominated by the tracking accuracy (*η*), the sample to detector distance (*L*_3_), the scanning step size (ν) and the number of modulator positions (M)^8^. One commonly used procedure to verify the angular sensitivity Δ*α* is to calculate the standard deviation of the wavefront gradient in empty space^24,25^. Following the same approach to quantify angular sensitivity, we have tracked the speckle displacement in empty space (marked as a rectangle in Fig. 2A) for one projection image to optimize the experimental parameters (M, ν and R). As shown in Fig. 4(D), the horizontal and vertical angular sensitivity decreased as the number of modulator positions was reduced. However, due to the subpixel accuracy of the advanced DIC algorithm, sufficient photon flux and good visibility of the speckles, the standard deviation of the marked region increased only from 0.15 μrad to 0.26 μrad even when the modulator number was reduced from 80 to 20. Hence, it is possible to achieve useful angular sensitivity by increasing the scanning step size in order to reduce the modulator position number, while maintaining a sufficient speckle scanning range. It should be noted that the angular sensitivity is deteriorated nearly by a factor of two when the modulator position number was reduced from 20 (B) to 10 (A), because the step size ν = 20 µm is close to the average speckle size.

A reconstructed phase slice of the volcanic rocks is shown in Fig. 4. The image quality in Fig. 4(B) (M = 20) is comparable that in Fig. 4(C) (M = 50) even though the image number has been reduced by a factor of 2.5. For the empty space marked in the square region in the reconstructed slice, the standard deviation is Δδ = 1.9 × 10^−8^ in Fig. 4(A) but is reduced to Δδ = 1.4 × 10^−8^ in Fig. 4(C). Although the phase sensitivity is compromised by the reduced number of images in Fig. 4(A) (M = 10), the different phases of the material within each rock can still be distinguished with significantly enhanced contrast and high spatial resolution. The total acquisition time for case B and C is 20 minutes and 50 minutes, respectively, while it is only 10 minutes for case A, achieved by cutting the modulator position number from 100 down to 10. According to the sampling theorem, the number of the projections will be dependent on the number of pixels in the direction perpendicular to the axis of rotation. For the speckle scanning technique, the spatial resolution is slightly worse than the detector’s effective pixel size because the neighboring pixels have to be selected for pixel-wise data analysis. Therefore, the number of projections N can be potentially further reduced to from 1200 down to 400 without significantly deteriorating the reconstructed spatial resolution^26^. Overall, it is feasible to further speed up the proposed fly-scan phase CT by optimizing the experimental parameters without significantly compromising the benefits of phase contrast imaging.

**Figure 5:**
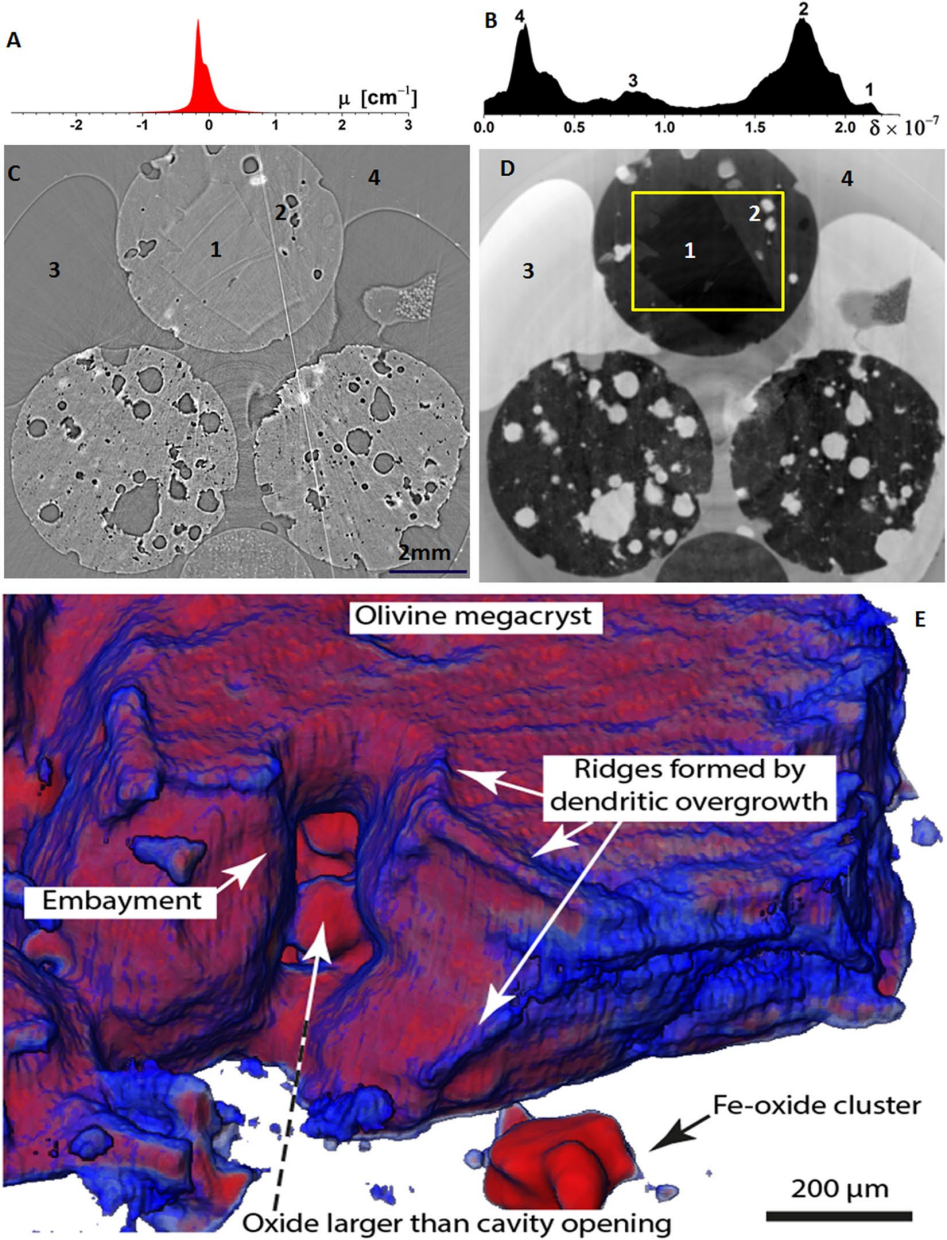
Reconstructed axial slices of picrite rock cores using transmission data (**C**) and phase data (**D)**. (**A**,**B**) are histograms of all the pixels in (**C**,**D**). (1) The olivine crystal, (2) glassy groundmass, (3) paraffin wax and (4) the air can be clearly identified in the phase signal data. (**E**) Volume rendering of phase contrast image focusing upon the edge of the large olivine crystal (box in **D**). Numerous textural features are made visible and allow 3D petrologic interpretation, such as the timing of crystal contact and growth stages.

As described above, results of the tomographic reconstruction for picrite rock at 53 keV using the fly-scan mode are presented in Fig. 5, where (A) and (B) are image histograms, and (C) and (D) are axial slices of the transmission and phase signal, respectively. The attenuation coefficients for different rock materials are similar at 53 keV, and the histograms of transmission slice in Fig. 5(A) show only one peak. Hence, different materials cannot be easily distinguished without substantial manual input and its attendant uncertainty. In Fig. 5(B), by comparison, multiple peaks are visible, each representing the real part of the refractive index of a different material. As highlighted in Fig. 5(D), the olivine crystal (1) can be easily distinguished from the glassy groundmass (2), whereas in Fig. 5(C) they are nearly indistinguishable. The paraffin wax (3) and the air (4) are also more strongly contrasted in the phase signal (Fig. 5(D)) than in the transmission signal (Fig. 5(C)).

Major improvements in image contrast that are observed in the corresponding phase CT image allow precise identification of the sample’s features. As shown in Fig. 5(E), numerous textural features such as an olivine megacryst, its embayment geometry and relationship to Fe-oxide clusters can be identified and quantified. This represents a breakthrough, because the rich information that can now be derived allows 3D petrologic interpretation such as the timing of crystal contact and growth stages^18^. Images that can distinguish between such components within a rock will unlock new avenues of volcanological study as well as improve existing research. For instance, being able to discriminate between different minerals, glasses etc. that have similar densities complements other advanced applications such as retrieving chemical information from certain minerals directly from the attenuation data^27^, because the 3D location and geometry of crystal edges can be quantified using the high-resolution, speckle scanning phase contrast tomography approach.

In order to penetrate materials with even higher atomic numbers or greater thickness, it is imperative to apply X-ray phase contrast CT using X-rays over 100 keV. Although steel wool can be used as modulator and achieve a higher absorption contrast than sandpaper, one of the main technical challenges that remain is the rapid drop in the visibility of an absorption pattern of steel wool whose strands are randomly oriented with respect to the X-ray direction (a tangle) with increasing photon energy. To circumvent this limitation we placed steel wool strands along the X-ray direction (a bundle).

**Figure 6:**
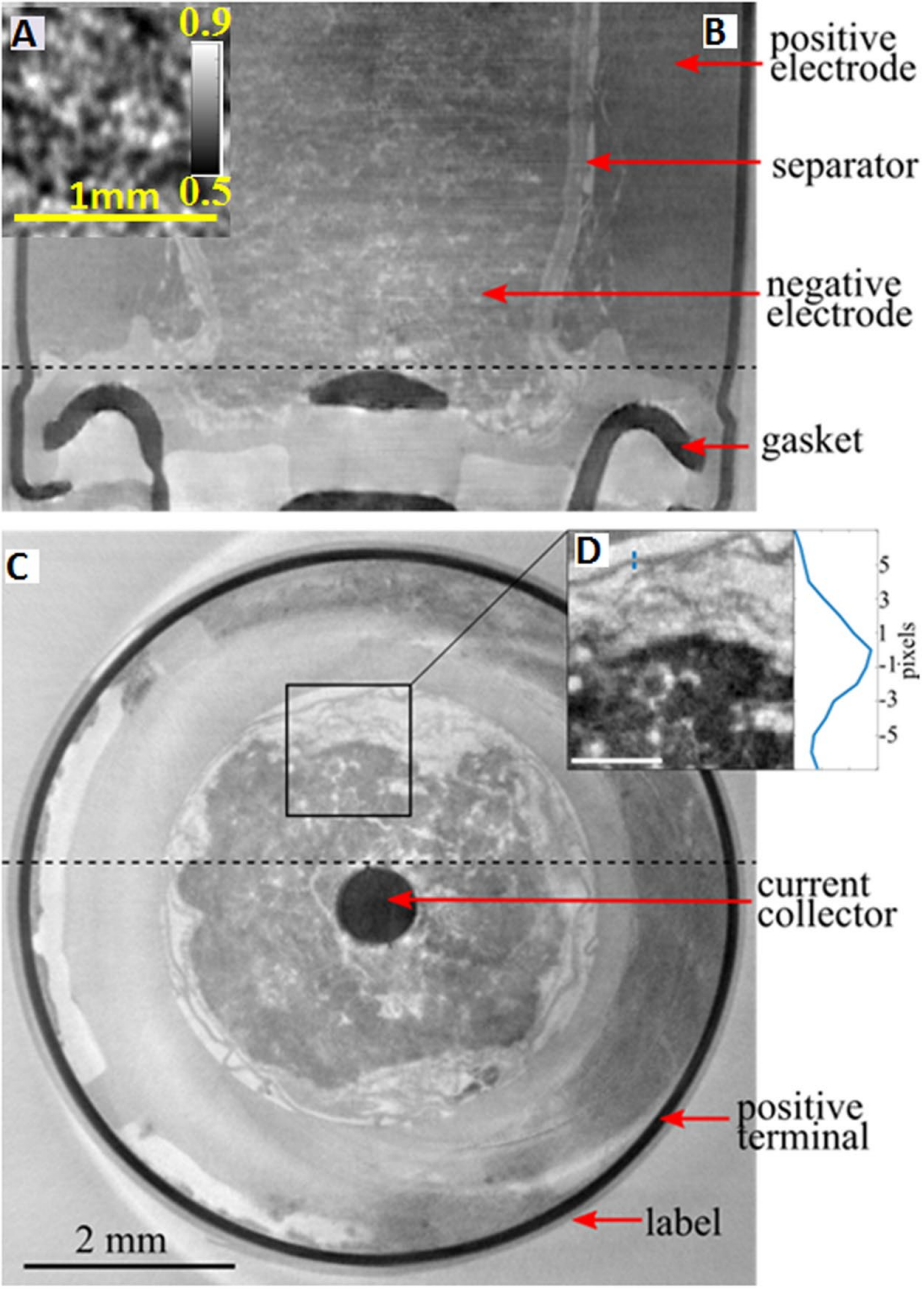
Reconstructed axial (**B**) and sagittal (**C**) slices of a battery sample from position indicated by dashed lines. (**A**) A raw speckle image after the flat field correction, and the visibility is 14% at 120 keV. (**D**) A magnified image of the small square region at bottom, shows coarse layers of particles from the separator and the negative electrodes. The scale bar at the bottom of the inset is 0.5 mm. The profile is taken from the inset where the blue line is marked.

Bundles of steel wool with individual strand lengths up to 100 mm were used here to partially block the high energy X-rays and still achieve useful absorption contrast. Special care was taken to mix the steel wool strands thoroughly to ensure that the wavefront modulator creates statistically uniform and locally randomly distributed structures over the illuminated area. For example, the amount of air space in the modulator should be optimized to gain sufficient transmission and suitable speckle size, which provides control over the inherent high frequency features of the transmitted speckle image. This control is affected simply by how well-packed the strands are in the bundle. Figure 6(A) shows a raw speckle image after optimizing the internal arrangement of steel wool. The visibility (defined as standard deviation normalized by average intensity) of the speckle pattern (within a 150 × 150 pixels window) generated by the modulator has reached up to 14% at 120 keV.

To demonstrate the potential applicability of the presented X-ray fly-scan tomography in energy science, further experiments were carried out by imaging a battery at 120 keV. The exposure time for each image during the tomography scan was 50 ms and each fly-scan tomography scan consisted of 1200 projections. The modulator was scanned horizontally with a step size of μ = 10 µm for M = 20 steps. A sagittal (B) and an axial (C) slice are shown in Fig. 6.

The layers of separator in the battery between the negative and positive electrodes can be easily recognized at 120 keV in the phase contrast image, while they would be difficult to distinguish by using conventional absorption CT since their attenuation coefficients at such high energy are very similar. The spatial resolution for this case is about 16 µm with effective pixel size of *P* = 7.9 µm, and the inset (D) clearly shows the thickness of the separator layer is ~35 µm. The quantitative phase CT method at such high energy will support future studies of heavy or thick material for advanced research in geosciences, civil engineering and material science. Since radiation doses are dramatically reduced above 100 keV, the fast phase CT can also be beneficial for biomedical application.

Compared to the standard step-scan scheme, the fly-scan method presented in this paper improves the data acquisition efficiency by a factor of 14. The data acquisition time has then been further reduced (5–10 times) by optimizing the scanning parameters. Consequently, the total acquisition time for one phase CT image has been reduced from tens of hours to tens of minutes.

By applying the speckle scanning technique to X-ray phase tomography, better spatial resolution has been attained than in the speckle tracking mode. Furthermore, the method has been extended into previously challenging X-ray energies up to 120 keV. The significant improvement in image contrast from the retrieved phase CT has facilitated precise identification of complex materials that was at worst impossible and at best impractical using conventional absorption CT.

Since the proposed method uses only inexpensive materials with random structures, the challenging fabrication of sophisticated optics for high energy X-rays is avoided and an easy implementation to a wide range of fields is promised. Although the proposed technique has been demonstrated with synchrotron radiation, it can be potentially implemented with laboratory X-ray micro-focus sources, thus permitting even wider application. It should be noted that this fly-scan scheme can also be used for other imaging techniques, such as grating interferometry or edge illumination, which require scanning optics for phase contrast tomography.

Moreover, white or pink beam can be used for fast phase tomography because it offers a flux over one magnitude higher than monochromatic beam would do^28^. Recently, new-generation high-speed cameras (a few hundred Hz up to thousands of Hz) and rotation stages have been employed for super-fast X-ray phase radiography and tomography^26,29,30^. By synchronizing a multi-axis motion stage with a camera and with a Zebra box, the speed of a fly-scan can be dramatically improved by moving both modulator and sample simultaneously and continuously. Overall, the data acquisition time of fly-scan tomography can be further reduced with a brighter source, faster tomographic hardware and multi-axis fly-scans.

We conclude that the speckle scanning technique combined with the fly-scan scheme is a promising tool for providing phase contrast CT with high speed. In turn, such fast phase tomography is poised for applications in dynamic experiments and underpins the investigation of active processes that operate at these rates, such as magmatic crystal settling and deformation in metal alloys.

## Methods

The experiment was performed at Diamond Light Source’s I12 beamline. Monochromatic X-ray beam produced by a double Laue crystal monochromator at 53 keV (and 120 keV) was used for various samples. The wavefront modulator was prepared with specially arranged steel wool (see main text) and mounted on a precision linear motorized stage. The distance between the modulator and the source *L*_1_ was about 50.0 m. The sample was placed *L*_2_ = 1.0 m downstream of the modulator. The distance between the sample and the detector was set to the maximum value *L*_3_ = 2.0 m to improve the phase sensitivity. The speckle pattern was recorded with an imaging camera featuring a field of view of 8 mm × 7 mm (and 20 mm × 17 mm using a different module) and an effective pixel size of *P* = 3.5 µm (and *P* = 7.9 µm).

The fly-scan mode was implemented and controlled with a ZEBRA system, which has been developed by the Controls Group at DLS^31^. The rotation of the sample for tomography was performed as a single command, moving the sample from −5° to 185° and programming the trigger signal to be raised as the stage passed zero, and at each increment of 0.15°. The wavefront modulator was then moved to its next position, and the trigger program was repeated so that images were again captured at zero degrees and at each selected increment. In this way, the tomography rotation is performed in between modulator steps, saving all the time overhead of moving and stopping the rotation stage. The time overhead of motion will be only M steps of the modulator, and the corresponding operations of returning the tomography stage back to zero. In this case, the proposed fly-scan method took 64 s to finish one tomography scan including the rewinding time of the rotation stage. The total acquisition time with N = 1200 projections, exposure time t = 5 ms, and modulator position M = 100 for the fly-scan scheme was less than 2 hours (T = 108 min).

Once the proposed fly-scan tomography scan was applied to the sample at different wavefront modulator positions, the collected speckle images for each sample were then reorganized in the sequence according to the modulator positions for each projection. It may be noted that for the one dimensional (1D) speckle scanning technique, the recorded pattern translates not only along the scanning direction but also along the horizontal direction after inserting the sample due to the 2D nature of the random pattern. The speckle pattern for each pixel is generated by combining the nearby pixels with the series of data along the scanning direction. A sub-pixel registration DIC algorithm is then applied between the speckle image with and without sample present in the beam, namely sample speckle signal and reference speckle signal^32^. The maximum of the cross-correlation coefficient can be precisely located for each pixel position. The speckle displacement (ξ^*x*^, ξ^*y*^) induced by the sample is related to the coordinate of the maximum of the cross-correlation coefficient. The wavefront gradient for the scanning direction and the orthogonal direction can then be calculated from the geometric magnification, the speckle displacement, the scanning step size and detector’s pixel size. The present horizontal scan mode obeys the relationship

1 $$\{\begin{array}{lllll}\frac{\partial \Phi (x,y)}{\partial x} & = & \frac{2\pi }{\lambda }{\alpha }^{x}(x,y) & \approx  & \frac{2\pi }{\lambda }\frac{{\xi }^{x}\nu ({L}_{1}+{L}_{2}+{L}_{3})}{{L}_{1}{L}_{3}}\\ \frac{\partial \Phi (x,y)}{\partial y} & = & \frac{2\pi }{\lambda }{\alpha }^{y}(x,y) & \approx  & \frac{2\pi }{\lambda }\frac{{\xi }^{y}P}{{L}_{3}}\end{array}.$$

where *v* is the piezo scanning step size of the modulator, *L*_1_, *L*_2_ and *L*_3_ represent the distances between X-ray source, sample, modulator and detector, α^*x*/*y*^ is the refraction angle in *x* or *y* direction, and Φ is the phase shift.

Thereafter, the phase shift Φ induced by the sample can then be obtained from the derived phase gradients by using the Fourier transform relation between a function and its derivative as follows^16^.

2 $${\rm{\Phi }}(x,y)=\frac{2\pi }{\lambda }{{\rm{F}}}^{-1}=[\frac{{\rm{F}}[{\alpha }^{x}(x,y)+i{\alpha }^{y}(x,y)](m,n)}{2\pi i(m+in)}](x,y)$$

where F^−1^ (F) is the inverse (forward) Fourier operation and (*m*, *n*) are the variables in the Fourier space corresponding to the real space variables (*x*, *y*)^16^.

Speckle data processing for each projection was performed with a compiled Matlab code, which was run using parallel processing distributed over 100 nodes of the DLS computation cluster. It took about one hour to process all of the 1200 datasets using a region of interest of 2560 × 1000 pixels. Since the produced phase shift Φ is proportional to the line integral of the refractive index decrement δ, it can be directly used in phase tomography reconstruction with filtered backprojection reconstruction methods^33^. Since the phase gradient represents the line integral of partial derivatives of the object’s refractive index decrement, it has also been used for the phase reconstruction for comparison. According to the fundamentals of computed tomography reconstruction, the refractive index decrement δ can be written as

3 $$\{\begin{array}{rcl}{\rm{\Phi }} & = & \frac{2\pi }{\lambda }\int \delta (z)dz\\ \int {\alpha }^{x}(x,y)dx & = & \int \delta (z)dz\end{array}$$

Both absorption coefficient μ and refractive index decrement δ have been reconstructed with Savu^34^, which is a tomography data processing tool developed in the Data Analysis Group at DLS. The Ram-Lak filter was applied for both absorption and phase reconstruction if the phase shift projection images were used, while the Hilbert filter was chosen for the phase gradient case^35^.
